# Early weight gain trajectories in first episode anorexia: predictors of outcome for emerging adults in outpatient treatment

**DOI:** 10.1186/s40337-021-00448-y

**Published:** 2021-09-14

**Authors:** A. Austin, M. Flynn, K. L. Richards, H. Sharpe, K. L. Allen, V. A. Mountford, D. Glennon, N. Grant, A. Brown, K. Mahoney, L. Serpell, G. Brady, N. Nunes, F. Connan, M. Franklin-Smith, M. Schelhase, W. R. Jones, G. Breen, U. Schmidt

**Affiliations:** 1 Department of Psychological Medicine, King’s College London, Institute of Psychiatry, Psychology, and Neuroscience, 16 De Crespigny Park, London, UK; 2 School of Health in Social Sciences, University of Edinburgh, Edinburgh, UK; 3 South London and Maudsley NHS Foundation Trust, London, UK; 4 Maudsley Health, Abu Dhabi, UAE; 5 Sussex Partnership NHS Foundation Trust, Brighton, UK; 6 North East London NHS Foundation Trust, London, UK; 7 Division of Psychology and Language Sciences, University College London, London, UK; 8 Central and North West London NHS Foundation Trust, London, UK; 9 Leeds and York Partnership NHS Foundation Trust, Leeds, UK; 10 Department of Social, Genetic & Developmental Psychiatry, King’s College London, London, UK

**Keywords:** Anorexia nervosa, Eating disorder, Early intervention, Outpatient, Treatment, Growth mixture modelling

## Abstract

**Background:**

Early response to treatment has been shown to be a predictor of later clinical outcomes in eating disorders (EDs). Specifically, early weight gain trajectories in anorexia nervosa (AN) have been shown to predict higher rates of later remission in inpatient treatment. However, no study has, as of yet, examined this phenomenon within outpatient treatment of first episode cases of AN or in emerging adults.

**Methods:**

One hundred seven patients with AN, all between the ages of 16 and 25 and with an illness duration of < 3 years, received treatment via the first episode rapid early intervention in eating disorders (FREED) service pathway. Weight was recorded routinely across early treatment sessions and recovery outcomes (BMI > 18.5 kg/m^2^ and eating psychopathology) were assessed up to 1 year later. Early weight gain across the first 12 treatment sessions was investigated using latent growth mixture modelling to determine distinct classes of change. Follow-up clinical outcomes and remission rates were compared between classes, and individual and clinical characteristics at baseline (treatment start) were tested as potential predictors.

**Results:**

Four classes of early treatment trajectory were identified. Three of these classes (*n* = 95), though differing in their early change trajectories, showed substantial improvement in clinical outcomes at final follow-up. One smaller class (*n* = 12), characterised by a ‘higher’ start BMI (> 17) and no early weight gain, showed negligible improvement 1 year later. Of the three treatment responding groups, levels of purging, depression, and patient reported carer expressed emotion (in the form of high expectations and low tolerance of the patient) determined class membership, although these findings were not significant after correcting for multiple testing. A higher BMI at treatment start was not sufficient to predict optimal clinical outcomes.

**Conclusion:**

First episode cases of AN treated via FREED fit into four distinct early response trajectory classes. These may represent subtypes of first episode AN patients. Three of these four trajectories included patients with substantial improvements 1 year later. For those in the non-response trajectory class, treatment adjustments or augmentations could be considered earlier, i.e., at treatment session 12.

## Plain English summary

A key feature of anorexia nervosa (AN) is an unhealthily low body weight. Previous studies show that more weight gained early in inpatient treatment leads to better outcomes. This study tried to see if this was also true for outpatients receiving treatment for the first time. All participants were emerging adults between the ages of 16 and 25 who had been ill for less than 3 years. Weight was recorded across the first 12 weekly treatment sessions. Statistics showed that the patients fit roughly into four different groups in early treatment, each with different starting weights and rates of weight gain in the first 12 treatment sessions. The group a patient belonged to could sometimes be predicted by vomiting behaviours, level of depression, and patients’ perception of parental tolerance and expectations at the start of treatment. Out of the four groups, three did relatively well 1 year later, but one small group of patients did not. This small group had a higher starting weight than many of the other groups but did not gain any weight across the first 12 sessions. These patients could benefit from a change or increase in the amount or intensity of treatment after the first 12 treatment sessions

## Introduction

Outpatient psychological therapies for adults with anorexia nervosa (AN) are associated with modest improvement in body mass index (BMI) and other outcomes, and there is no evidence for superiority of any specific approach. Such findings highlight the need to further develop and improve treatments [1]. A better understanding of individual characteristics, moderators, and trajectories in treatment is crucial in order to tease apart what works best for whom (i.e., to develop a precision medicine approach), and also to reduce unsuccessful treatment attempts [2].

Early response to treatment has been identified as a possible predictor of later clinical outcomes in eating disorders (EDs) [3, 4], i.e., those who have early symptom reduction after starting treatment are likely to have better outcomes at end of treatment and at later follow-ups. Recent studies evaluating early treatment response in EDs have adopted a latent growth modelling approach [5–7]. The purpose of this approach is to identify meaningful subgroups of patients with distinct growth (recovery) trajectories within a larger heterogeneous patient group [8]. Specific to AN, weight gain during early treatment has been shown to predict later rates of remission [9]. Application of a latent growth modelling approach to the treatment of AN, with the identification of these early weight gain subgroups, and individual and clinical characteristics that predict membership to these groups, may allow clinicians to determine the prognosis of patients and consequently tailor treatment to their needs.

Previous studies looking at treatment response in AN using a latent growth modelling approach have largely focused on full and partial hospitalisation settings. In a study of 102 adolescents and young adults with AN who were partially hospitalised, Berona, Richmond, and Rienecke found three distinct early weight gain trajectories: a slow, a moderate, and a rapid class. The rapid weight gain class membership was predicted by three characteristics at baseline (i.e., treatment start): the presence of compensatory behaviours, lower parental expressed emotion, and the absence of a comorbid mood disorder [10]. Similarly, in an inpatient sample, Makhzoumi et al. found that a rapid weight gain trajectory was associated with regular restriction, bingeing, and purging, and further determined that a faster weight gain trajectory was associated with greater weight restoration at follow-up [11].

Most recently, Wade et al. investigated the trajectories of early weight gain in AN during outpatient treatment [12]. Four distinct trajectories were found, and the class with the highest weight gain over the early treatment period had the greatest rates of later remission. Results also showed that a class with higher BMI at treatment start did not automatically have better clinical outcomes than a class with a low BMI at treatment start [12]. This supports the need for the consideration of growth patterns rather than only severity of BMI at baseline for appropriate treatment selection.

To date, no studies have specifically assessed early weight gain trajectories for outpatients experiencing their first episode of AN, i.e., in a treatment naïve state. This is important to assess as first episode AN patients tend to have a more favourable treatment response compared to those with a more established illness [13, 14]. Thus, previous trajectory analyses in outpatient AN may not generalise to a first episode population.

In the current study we attempt to address this gap, with the aim to:

1. Investigate typical weight gain trajectory classes in outpatient treatment for first episode AN.
2. Evaluate baseline variables to determine if any predict membership of trajectory classes.
3. Evaluate outcome (remission) variables of each class.

## Methods

### Design

This study involves an analysis of weekly BMI and ED behavioural symptom data, logged weekly by clinicians during the multi-centre FREED-Up study. This study had a quasi-experimental pre-post design comparing 278 First Episode Rapid Early Intervention for Eating Disorders (FREED) patients to 224 treatment-as-usual controls, who were similar patients seen in the 2 years before FREED was introduced. The study and its findings are described in detail elsewhere [15, 16].

### Participants

Participants were consecutive referrals from four specialist ED centres in England. All were emerging adults who entered treatment for a first episode ED (illness duration < 3 years) between 2016 and 2018 and were between 16 and 25 years old at study enrolment. Patients were excluded if they needed an immediate inpatient admission, were pregnant, had a severe learning disability, or had a comorbid physical or mental disorder needing primary treatment (e.g., psychosis). One hundred and twenty-one met diagnostic criteria for DSM-5 AN or other specified feeding and eating disorder [17] at assessment and had a BMI < 18.5 at the start of treatment. Of these, 107 patients (88.4%) had symptom log data available, which constituted our final sample.

### Procedure

Details of the FREED service model and care pathway has been previously described [18–21]. In brief, FREED patients were given a phone call within 48 h of referral to screen for eligibility for the service, and to increase engagement. Patients potentially suitable for FREED were offered a clinical assessment adapted for FREED, taking a biopsychosocial, person-centred approach, with family involvement encouraged. The adapted assessment emphasised the importance of early intervention on ED-related changes to the brain and body. Patients were then allocated to treatment, with the aim of starting this within 2 weeks of assessment. Treatment was NICE-concordant [22], evidence based (e.g., ED focused cognitive behavioural [CBT-ED] or Maudsley Anorexia Nervosa Treatment for Adults [MANTRA]), tailored to the needs of emerging adults in early-stage illness, and typically lasting between 20 and 30 individual sessions. Developmentally informed adaptations included a focus on early dietetic involvement and nutritional change, reduction of any unhelpful/excessive social media and health-related app use, effective management of transitions (e.g., to university, in treatment), the developmental tasks of emerging adulthood and age-appropriate family involvement.

### Measures

#### Clinician symptom log

Therapist-recorded BMI and ED behaviour frequency at weekly therapy sessions.

#### Eating disorder examination questionnaire (EDE-Q)

The EDE-Q [23] is a 28-item measure which captures the frequency and severity of ED behaviours over the past 28 days. It provides a score on four subscales (dietary restraint, eating concerns, shape concerns, and weight concerns) as well as a global score. A total global score > 2.8 suggests a clinical ED. [24] The EDE-Q also measures the frequency of binge and compensatory behaviours over the last 28 days [23].

#### Depression anxiety stress scale-21 (DASS-21)

The DASS-21 [25] is a 21-item screener which captures mood over the past week. It contains subscales for depression, anxiety, and distress, as well as a global score.

#### Clinical impairment assessment (CIA)

The CIA [26] is a 16-item measure used to evaluate psychosocial impairment from an ED. It covers four domains: mood and self-perception, cognitive function, work performance, and interpersonal function.

#### Level of expressed emotion (LEE)

The LEE [27] is a 60-item true or false questionnaire used to evaluate the perception of expressed emotion of one’s most influential relationship. It includes subscales for attitude toward illness, emotional response, intrusiveness, and low tolerance/high expectations.

### Analysis

#### Derivation of latent classes

The rate of change in weekly BMI over the first 12 therapy sessions (the approximate halfway point) was used to determine latent class membership in the current study. Patients who took a break for more than 30 days between treatment sessions (e.g., for exams, holidays) during the first 12 weeks only had data included up to the point of absence. Latent growth mixture modelling (LGMM) was used, which categorises individuals with similar patterns of longitudinal change into subgroups while also allowing for individual variation [28]. The optimal number of subgroups was informed by fit statistics including the Akaike Information Criteria (AIC), the Bayesian Information Criteria (BIC), and the sample-size adjusted Bayesian Information Criteria (aBIC), with lower absolute values indicating a better model fit. Entropy, or the separateness of the classes, was also evaluated in each model, with a value above 0.8 suggesting good separation [29]. Finally, the Vuong-Lo-Mendell-Rubin likelihood ratio test (VLMR-LRT) and the adjusted Lo-Mendell-Rubin likelihood ratio test (adjusted LRT) were used to compare a model with X classes to a model with X-1 classes, with a *p* value < 0.05 indicating that a model with X classes fits better the model with X-1 classes. LGMM was first conducted with a one class model, increasing up to a five-class model. Analysis was performed in Mplus version 8.4 (Muthén & Muthén, 2019).

#### Latent classes and clinical characteristics

Latent classes were compared on baseline variables (predictors) and 12-month follow-up variables (outcomes) using a 3-step approach as recommended by Herle et al. [30]. One way analysis of variance (ANOVA) was used to compare trajectory classes on continuous variables (e.g., EDE-Q score) while chi-squared and Fisher’s exact tests were used for categorical variables (e.g., ethnicity). Significant findings were then subject to post-hoc testing to determine which classes differed. Binge, purge, and laxative use frequencies were zero-inflated and so groups were compared on the presence or absence of these behaviours. Remission was defined as BMI > 18.5 kg/m^2^ and an EDE-Q global score < 2.8 as suggested by Mond et al. [24]. For participants with missing data at the 12-month follow-up, data from the 6-month timepoint were used. Analysis was done in SPSS version 26.

## Results

### Latent classes

**Fig. 1:**
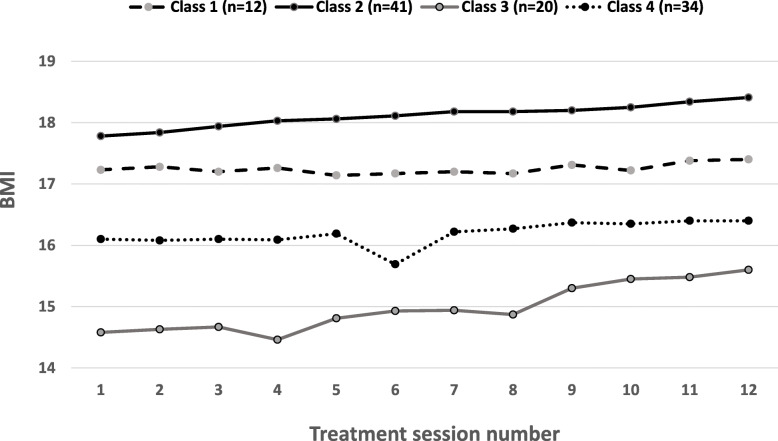
The selected four class model as best fits the data, here showing weight gain across early treatment sessions

Fit statistics from the latent class analyses are presented in Table 1. One to five class solutions were tested, with entropy (i.e., separateness of the classes) increasing with each subsequent analysis. As recommended, the best fitting solution was determined by both fit statistics and existing findings/previous theory. Following previous evidence, we anticipated a three to four class solution [10–12, 31]. Two of the three fit statistics (AIC and aBIC) were lowest for the four-class solution. Thus, a four-class solution best fit the data, as is presented in Fig. 1. This includes one class starting with a higher BMI, making little change across the first 12 therapy sessions (*higher, stable*). A second class also starts at a higher BMI but makes steady, moderate gains across this same time period (*higher, moderate*). A third class starts treatment with a very low BMI but makes large gains in early treatment (*low, rapid*). Finally, a fourth class begins at a moderate BMI and makes little early change (*medium, stable*).

**Table 1:** Fit statistics for latent growth mixture modelling

Classes	# free parameters	AIC	BIC	aBIC	Entropy	^a^LRT P
1	19	1602.64	1653.60	1593.56	–	–
2	23	1593.16	1654.85	1582.17	0.76	0.32/0.34
3	27	1589.36	1661.78	1576.47	0.87	0.34/0.36
4	31	1584.38	1667.53	1569.58	0.88	0.48/0.49
5	35	1588.67	1682.55	1571.96	0.90	0.24/0.24

*aBIC* sample-size adjusted Bayesian information criteria, *AIC* Akaike information criteria, *BIC* Bayesian information criteria

^a^*LRT* likelihood ratio tests (Vuong-Lo-Mendell-Rubin likelihood ratio test; adjusted Lo-Mendell-Rubin adjusted LRT test) quantify specific comparisons between the model of interest and a model with one fewer class, C-1

### Baseline predictors

Baseline characteristics of each latent class can be seen in Table 2. Participants in Class 2 (*high, moderate*) were significantly more likely to report higher scores on depression than Class 3 (*low, rapid*) and higher patient reported carer expressed emotion (low tolerance/greater expectations) compared to participants in Class 4 (*medium, stable*). Class 2 also had the highest rates of binge, purge, and laxative use behaviours at baseline, although only the presence of purging significantly predicted membership into Class 2 compared to Class 3 (*low, rapid*). These baseline findings are non-significant after a Bonferroni correction.

**Table 2:** Baseline characteristics (predictors) of each latent class, with mean and standard deviation

Variable	Class1, *n* = 12 (high, stable)	Class2, *n* = 41 (high, moderate)	Class3, *n* = 20 (low, rapid)	Class4, *n* = 34 (medium, stable)	Group comparison
Age (years)	19.50 (1.45)	19.22 (2.15)	20.10 (2.08)	20.15 (2.32)	F(3,103) = 1.46, *p* = 0.23
DUED (months)	14.73 (9.19)	16.98 (12.17)	16.05 (9.01)	16.88 (10.83)	F(3,101) = 0.15, *p* = 0.93
Baseline BMI	17.91 (0.66) ^1^	17.66 (0.97) ^1^	14.74 (0.65) ^2^	16.23 (0.72) ^3^	**F(3,87) = 3.44,*p*** **= 0.02** ^a^
Ethnicity, n (%)					X^2^ (3) = 4.57, *p* = 0.21
White	9/11 (81.8%)	28/36 (77.8%)	17/19 (89.5%)	22/32 (68.8%)	
BAME	2/11 (18.2%)	8/36 (22.2%)	2/19 (10.5%)	10/32 (31.3%)	
Occupation, n (%)					X^2^ (6) = 3.64, *p* = 0.74
Student	10/12 (83.3%)	28/41 (68.3%)	12/20 (60.0%)	21/34 (61.8%)	
Employed	1/12 (8.3%)	7/41 (17.1%)	6/20 (30.0%)	7/34 (20.6%)	
Unemployed	1/12 (8.3%)	6/41 (14.6%)	2/20 (10.0%)	6/34 (17.6%)	
Home, n (%)					X^2^ (3) = 6.84, *p* = 0.08
With family	3/10 (30.0%)	29/40 (72.5%)	10/19 (52.6%)	21/34 (61.8%)	
Other	7/10 (70.0%)	11/40 (27.5%)	9/19 (47.4%)	13/34 (38.2%)	
EDE-Q	4.42 (1.21)	3.95 (1.25)	3.62 (1.28)	3.33 (1.57)	F(3,103) = 2.39, *p* = 0.07
Binge	6/12 (50.0%)	21/41 (51.2%)	9/20 (45.0%)	9/34 (26.5%)	X^2^ (3) = 5.18, *p* = 0.16
Purge	3/12 (25.0%)	15/41 (36.6%) ^1^	1/20 (5.0%) ^2^	5/34 (14.7%)	**Fisher’s exact = 9.23,*p*** **= 0.02** ^a^
Laxative	2/12 (16.7%)	9/41 (22.0%)	3/20 (15.0%)	1/34 (2.9%)	Fisher’s exact = 6.26, *p* = 0.08
DASS - 21	32.83 (14.06)	35.17 (14.04)	27.45 (9.90)	33.50 (14.62)	F(3, 103) = 1.48, *p* = 0.23
Depression	11.92 (4.98)	13.02 (5.61) ^1^	8.80 (4.51) ^2^	11.24 (5.56)	**F(3, 103) = 2.86,*p*** **= 0.04** ^a^
Anxiety	8.42 (4.70)	9.49 (5.71)	5.90 (4.04)	9.24 (5.38)	F(3,103) = 2.35, *p* = 0.08
Stress	12.50 (5.90)	12.66 (4.54)	12.75 (4.46)	13.03 (5.18)	F(3,103) = 0.05, *p* = 0.99
CIA	34.83 (8.6)	32.20 (10.86)	32.20 (9.68)	29.76 (11.03)	F(3,103) = 0.79, *p* = 0.50
LEE	13.58 (13.91)	18.26 (11.99)	15.1 (11.52)	11.97 (8.45)	F(3, 103) = 2.08, *p* = 0.53
Intrusiveness	4.92 (3.73)	5.80 (3.88)	4.95 (3.62)	4.64 (2.74)	F(3, 102) = 0.74, *p* = 0.53
Emotional response	2.58 (4.14)	4.50 (3.60)	3.95 (3.89)	2.74 (3.51)	F(3, 102) = 1.77, *p* = 0.16
Attitude toward illness	2.58 (2.61)	3.50 (2.72)	2.60 (2.52)	2.09 (1.48)	F(3, 102) = 2.32, *p* = 0.08
Tolerance/expectations	3.50 (4.08)	4.93 (3.77) ^1^	3.60 (3.56)	2.50 (2.54) ^2^	**F(3, 102) = 3.12,*p*** **= 0.03** ^**a**^

*BAME* Black, Asian, and minority ethnic, *CIA* Clinical Impairment Assessment, *DASS-21* Depression, Anxiety, and Stress Scale, 21-item version, *DUED* duration of untreated eating disorder, *EDE-Q* Eating Disorder Examination Questionnaire, *LEE* Level of Expressed Emotion Scale

^1,2,3^ Different superscripts indicate significant differences between the classes. For example, Class 2 and Class 3 have significantly different rates of depression. ^a^ Non-significant after Bonferroni correction

### Recovery outcomes

Outcome characteristics of each latent class are presented in Table 3.

**Table 3:** Outcome characteristics of each latent class

Variable	Class1, *n* = 12 (high, stable)	Class2, *n* = 41 (high, moderate)	Class3, *n* = 20 (low, rapid)	Class4, *n* = 34 (medium, stable)	Group comparison
Follow-Up BMI	17.56 (1.69) ^1^	19.32 (1.73) ^2^	18.03 (2.95)	18.17 (1.49)	**F(3,87) = 3.44,*p*** **= 0.02** ^a^
Follow-Up BMI > 18.5	4 /11 (36.4%)	20/32 (62.3%)	7/16 (43.8%)	15/32 (46.9%)	X^2^ (3) = 3.18, *p* = 0.39
BMI change	0.38 (1.68) ^1^	1.57 (1.80) ^1^	3.40 (2.95) ^2^	1.95 (1.55)	**F(3,87) = 5.66,*p*** **< 0.001**
Follow-Up EDE-Q	2.50 (1.48)	2.64 (1.88)	2.28 (1.80)	1.91 (1.41)	F(3,77) = 0.96, *p* = 0.42
Follow-Up EDE-Q < 2.8	6/10 (60.0%)	19/29 (65.5%)	9/14 (64.3%)	23/29 (79.3%)	X^2^ (3) = 2.14, *p* = 0.59
EDE-Q change	−1.75 (1.31)	−1.51 (1.67)	− 1.30 (1.75)	−1.44 (1.24)	F(3,69) = 0.19, *p* = 0.90
Completed treatment	11/12 (91.7%)	28/41 (68.3%)	13/20 (65.0%)	29/34 (85.3%)	Fisher’s Exact = 5.61, *p* = 0.14
Intensive treatment ^b^	–	2/41 (4.9%)	4/20 (20.0%)	3/34 (8.8%)	Fisher’s Exact = 0.18, *p* = 0.15
Remission at Follow-up	1/10 (10.0%)	13/30 (43.3%)	5/16 (31.3%)	10/30 (33.3%)	Fisher’s Exact = 3.84, *p* = 0.28

*BMI* Body mass index, in kg/m^2^, *EDE-Q* Eating Disorder Examination Questionnaire

^1,2,3^Different superscripts indicate significant differences between the classes. For example, Class 1 and Class 2 have significantly different follow-up BMI

^a^Non-significant with Bonferroni correction; ^b^ Intensive treatment refers to stepped up care into day or inpatient during the 12-month follow-up period

Follow-up BMI at 12 months was higher for Class 2 (*high, moderate*) compared to Class 1 (*high, stable*), although this was no longer significant after a Bonferroni correction to account for multiple testing.

The trajectory with the lowest starting BMI (Class 3: *low, rapid)* had significantly greater BMI change between treatment start and 12-month follow-up than Class 1 (*high, stable*) and Class 2 (*high, moderate*).

No other significant differences between classes were found. Class 1 (*high, stable*) had the lowest proportion of weight restored participants (BMI > 18.5 kg/m^2^) and the lowest rates of full remission (10%), although these finding were not statistically significant.

## Discussion

Our first aim was to investigate the typical trajectory classes of early weight gain across outpatients with first episode AN. Fit statistics suggested that a four-class solution best fit the data. This consisted of 1) a class of patients with relatively high BMI (> 17 kg/m^2^) at treatment start and stable weight (i.e., no improvement) across early treatment (*high, stable)*, 2) a class with relatively high BMI at treatment start but with moderate weight gains (about half a BMI point) across early treatment (*high, moderate*), 3) a class with a medium starting BMI relative to other classes but with little improvement over early treatment (*medium, stable*), and 4) a class with extremely low BMI (< 15 kg/m^2^) and fast improvement across early treatment (*low, rapid*). This is similar to Wade et al., who found four classes with similar start BMIs (two with higher values, one medium, and one low) [12].

The second aim was to determine whether any characteristics may predict class membership. Those in Class 2 (*high, moderate*) were more likely to report higher levels of depression than Class 3 (*low, rapid*) and higher reported parental expressed emotion (greater expectations/lower levels of tolerance) compared to those in Class 4 (*medium, stable*). A previous study by Berona et al. found that the presence of a comorbid mood disorder and higher levels of parental expressed emotion were predictive of slower early weight gain [10]. However, it is still unclear exactly how depression/mood and parental expressed emotion contribute to trajectory change classes in first episode AN. For example, depression scores were more severe in a group (Class 2) with higher starting BMI and moderate trajectory improvements and lower in a group (Class 3) with poorer starting BMI and rapid trajectory improvements. Future research will need to tease apart the relationship between these predictive variables and their relationship to intercept (start BMI) and slope (trajectory change).

Class 2 (*high, moderate*) also had the highest rates of binge, purge, and laxative use behaviours at baseline, although only the presence of purging predicted membership into Class 2 compared to Class 3 (*low, rapid*). Previous work has found compensatory behaviours to be predictors of more rapid weight gain trajectories in early inpatient treatment [10, 11]. However, it is difficult to directly compare our results to this previous work as these studies focused on the rate of weight gain irrespective of a patient’s starting weight (i.e., all patients started at ‘zero’). A transdiagnostic study by Espel-Huynh et al. found that the presence of vomiting at baseline was more common in patients with a rapid response trajectory in early treatment as measured by ED symptoms and emotional functioning [5]. As such, compensatory behaviour should be considered a specific variable of interest in any future treatment response trajectory studies. Overall, after correcting for multiple testing, there were no robust baseline predictors of later clinical outcomes.

Our third aim was to compare classes by later clinical outcomes. Three of the four classes achieve substantial improvements at 12-months. For the classes with a lower starting BMI, these improvements were ‘propped up’ by higher use of additional intensive treatments, although the difference in use of intensive treatments was not significantly different between classes. While these three classes had differing early treatment trajectories, i.e., some classes responding rapidly and others taking longer, all three achieved substantial and similar clinical outcomes. Conversely, those in Class 1 (*high, stable*) had the lowest rate of remission (10%) compared to the other classes (31–43%). This demonstrates that, similar to Wade et al.’s findings [12], a higher BMI at treatment start is not sufficient to predict later remission or even weight restoration in AN, and this seems to be even more pronounced in first episode cases.

One clinical implication of these results is the consideration of adjunct or alternative treatments for those with first episode AN. In our study, a small group (*n* = 12) of patients with a ‘relatively’ higher BMI (i.e., > 17) who do not gain weight over the first 12 sessions (Class 1: *high, stable*) had the poorest recovery rates at 12 months, at only 10%. It may be that a change or augmentation to therapy for a first episode patient is more suitable at the mid-point of treatment rather than simply carrying on ‘as is.’ This could include intensifying session frequency, increasing family involvement, or changing treatment setting (e.g., day treatment). Alternatively, adjunctive medications (e.g., antidepressants or olanzapine) focused on the ED or a comorbidity might be considered [32], or emerging treatments such as cognitive remediation therapy [33], or neuromodulation approaches [34].

A key limitation of this work is the relatively small sample size. This may impact the reliability of results, and as such, these findings should be considered exploratory. However, the sample size was above the minimum of 100 as recommend for LGMM [5, 35]. Additionally, while patients’ weight for BMI calculations was measured at each weekly treatment session by a clinician, all other variables were assessed by self-report. Data gathered by self-report rely on patient memory and insight, which may reduce validity. Finally, clinical outcome data were analysed using complete case analysis with 6-month outcomes substituted when 12-month outcomes were unavailable, which poses a risk for bias. Future research would ideally have longer and more complete follow-up data.

In conclusions, patients with first episode AN fit into four distinct trajectory classes, three of which had substantial weight gain at 12-months. Depression scores, the presence of purging, and perceived levels of parental/carer expressed emotion in the form of high expectations/low levels of tolerance were predictive of class membership. A higher BMI at treatment start was not sufficient to produce better weight restoration at 12-month follow-up. These results are exploratory in nature and should be interpreted with caution until larger studies can clarify findings.

## Data Availability

The datasets used and/or analysed during the current study are available from the corresponding author on reasonable request.

## Abbreviations

CBT-ED: Eating disorder focused cognitive behavioural therapy
DUED: Duration of untreated eating disorder
FREED: First episode rapid early intervention for eating disorders
LGMM: Latent growth mixture modelling
MANTRA: Maudsley model of anorexia nervosa treatment for adults
NICE: National Institute for Health and Care Excellence
